# Bendductor—Transformer Steel Magnetomechanical Force Sensor

**DOI:** 10.3390/s21248250

**Published:** 2021-12-10

**Authors:** Przemysław Grenda, Monika Kutyła, Michał Nowicki, Tomasz Charubin

**Affiliations:** Warsaw University of Technology, Institute of Metrology and Biomedical Engineering, A. Boboli 8, 02-525 Warsaw, Poland; przemyslaw.grenda.stud@pw.edu.pl (P.G.); kutyla@jinr.ru (M.K.); tomasz.charubin@pw.edu.pl (T.C.)

**Keywords:** magnetoelastic effect, Villari effect, force sensor

## Abstract

In this paper, the design and investigation of an innovative force sensor, based on the Villari effect, is presented. The sensor was built from electrical steel, in a pressductor pattern, but working in bending load mode. The results of the experimental research allowed for the evaluation of transducer’s performance, mitigation of measurement hysteresis, and optimization of its functional parameters. Several issues have been examined, among them the selection of supply and measured signals, the measured values’ impact on measurement hysteresis, harmonic analysis, and the selection of proper current waveforms and frequencies. The proposed sensor is robust, made from inexpensive materials, and has high sensitivity, as compared to other magnetoelastic sensors. It has much higher stress sensitivity than other magnetoelastic sensors due to deformation mode. Based on the tests, its measuring range can be defined as 0.5–5 N with a near-linear characteristic, SNR of 46 dB, and 0.11 N uncertainty.

## 1. Introduction

Magnetoelastic sensors are one of heavily researched types of force sensors and are typically used for special applications [1]. They are known for their significant sensitivity [2], robustness [3], low temperature coefficients, and high measurement ranges [4]. Among the many presented magnetoelastic solutions are the most industrially widespread pressductors [5] with orthogonal magnetizing and sensing coils, frames with coaxial field and stress distribution [6], ring-cores in sideways stress (Mohri’s) [7], and orthogonal stress (Szewczyk’s) configurations [8], as well as numerous torque-sensing designs [9,10,11,12,13]. What is more, new and improved sensor configurations are still appearing [14].

**Figure 1:**
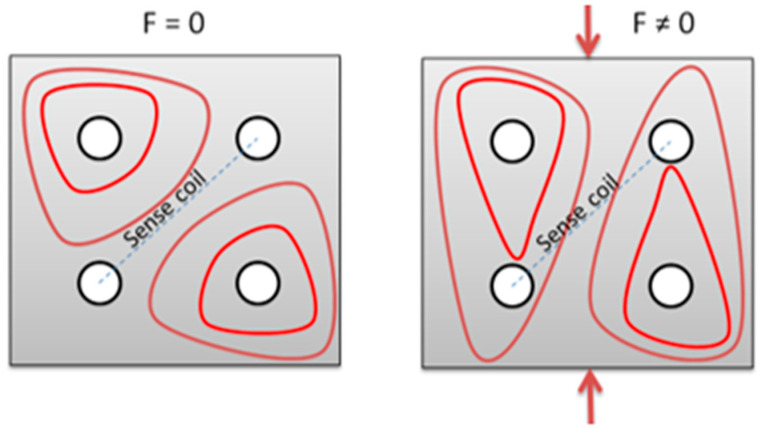
Distribution of magnetic field lines in the pressducer under the action of a non-zero compressive force [3,30].

Most of the available force sensors based on the inverse magnetomechanical effect (i.e., Villari effect) are applicable for the kN-MN ranges [15]. The most common pressductor has been in use since 1954 [5]. Its scheme of operation is presented in Figure 1.

A pressductor is built from a steel core, with four holes in which orthogonal magnetizing and measuring coils are made [5]. When alternating current is passed through the magnetizing coil, a magnetic field is created in the core. The lines of this field are arranged as shown in Figure 1, and are parallel with the measurement coil. Consequently, no current is induced in the measuring coils [5]. After the core is loaded with compressive force, the internal distribution of the field lines changes as a result of the Villari effect [16]. As a result of this change, an alternating current is induced in the measuring coils, the changes of which can be related to the compression force of the steel core.

The core of the pressductor is usually made of laminated electrical steel with compressive forces affecting on it [17]. The high-silicone electrical steel is typically used in power conversion as transformer cores [18,19,20]. It is also utilized in electrical machines such as electric motors, both AC and DC, and both as stators and rotors and, depending on the particular motor configuration, for electric vehicles as well [21,22,23]. Despite the significant magnetoelastic (Villari) effect [24,25,26,27], there are not many papers on these properties, even though they can be positive or detrimental for particular electric machine operation due to magnetoelastic effect induced anisotropy in the electric steel sheets, which is potentially more severe than other investigated effects such as texture [28] or magnetic aging [29]. What is more, there are even fewer works on utilizing this effect for sensor construction, with the original design for the pressductor being the lone commercially available solution [3].

However, the Villari effect occurs for the stresses acting on the core in any directions [3]. Therefore, the article below presents the operation of the sensor (bendductor) based on the forces resulting in shear stress (bending moment). Taking into account the distribution of stresses created after multiplication by the lever, the bending moment affects the core much faster than in the pressductor (or tensductor), changing the distribution of the magnetic field lines for forces from ~0.1 N.

M = F × r(1)

where M is the bending moment, F is the shear force, and r is the distance between the force and the point where the moment is calculated.

Thus, the main advantage of the bendductor over other magnetoelastic sensors is the possibility of small forces measurement, as most of the magnetoelastic technologies focus on high measurement ranges [1]. The advantage over existing metallic and semiconducting strain gauge sensors, most commonly used in small force measurements, is the ability to withstand harsh environments, a wide range of temperatures, higher resistance to external interferences, and high SNR (signal to noise) values which are typical for magnetoelastic technologies (especially for steel-based cores) [1,6]. Therefore the possible place of this sensor is in low-force, medium-accuracy, high reliability industrial applications, where the resistance to working conditions is of higher importance than other parameters.

## 2. Materials and Methods

The base of the bendductor is an electrical steel sheet core. The dimensions of the manufactured plate were 80 × 80 mm, with winding holes drilled in the rectangular 40 × 40 mm pattern.

**Figure 2:**
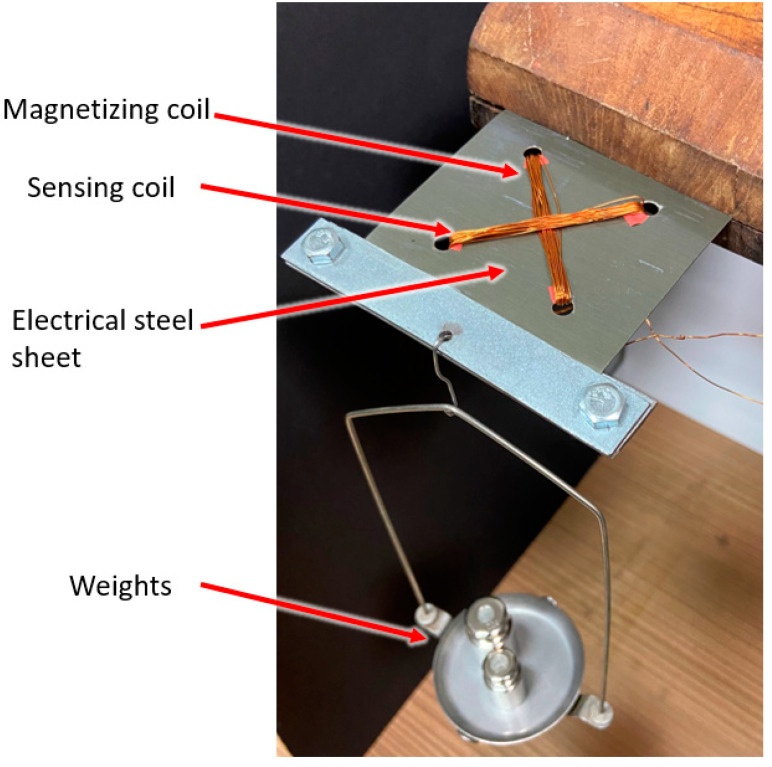
Force sensitive element—electrical steel sheet drilled and wound with orthogonal magnetizing and sensing windings in pressductor pattern.

**Figure 3:**
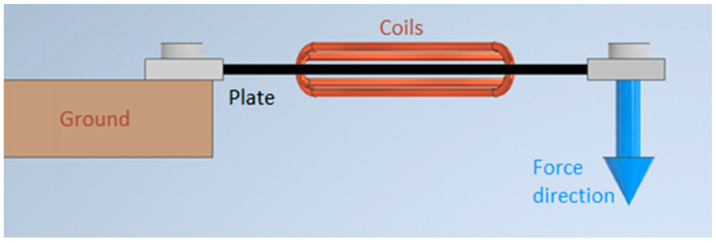
Plate loading scheme.

Through the prepared 4 × φ4 holes, respectively 20 measuring and magnetizing turns were threaded. The plate was closed with a housing made of four flat bars, which were used to load the sensor with a force. The board prepared for connection with electrical systems is shown in Figure 2 and its method of fixing and loading is shown in Figure 3.

**Figure 4:**
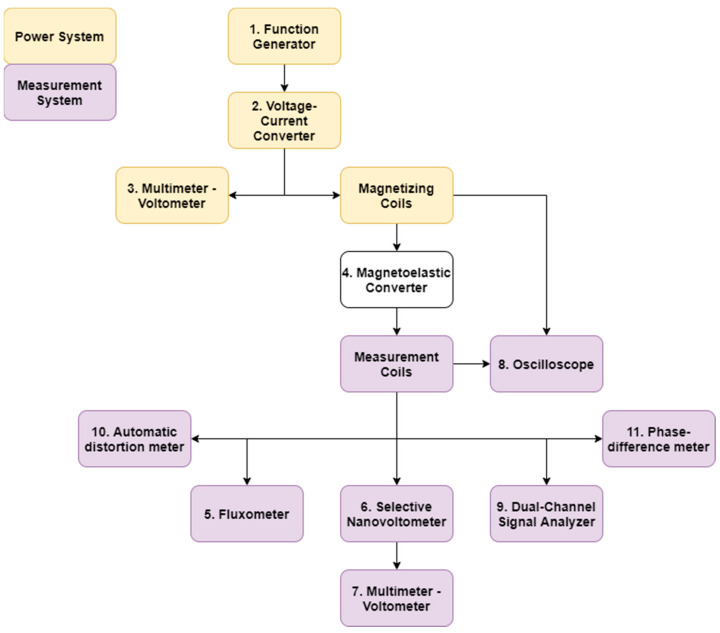
Diagram of the stand for measuring physical quantities other than voltage.

The coils magnetizing the plate can be supplied with alternating current of different amplitude, frequency, or waveform shape. The test station was suitable for power supply working in voltage or current control mode (Figure 4).

The first variant used the Siglent SDG1050 (1) function generator controlling ZOPAN Type PO-27 power generator (2). Driving voltage was measured with APPA 207 multimeter (3).

In the current-controlled mode, the power generator was substituted by voltage-current converter type Meratronik P334 (2). Driving current was measured with APPA 207 multimeter (3).

The measuring coil was connected to Unipan type 233 selective nanovoltmeter (6), which was tuned to operate at the frequency set by the power generator and operated as high-Q band-pass filter. Filtered signal was measured by Tonghui 1961 multimeter (7). A Schlumberger 5228 analog oscilloscope (8) was used to monitor the waveforms, which was set into a two-channel mode and the input and output from a selective nanovoltmeter were compared.

Beside the selective voltmeter, additional measurement equipment was employed for investigation of various signal parameters:

- LakeShore Model 480 Fluxmeter (5) set to measure magnetic flux density in sensor core;
- F2-16 phase difference meter (11) comparing input and output signals in the transducer;
- PMZ-12 nonlinear distortion meter measuring THD (total harmonic distortion) of the output signal;
- Bruel and KJAER type 2034 dual-channel spectrum analyzer used for measurements of signal harmonics.

**Figure 5:**
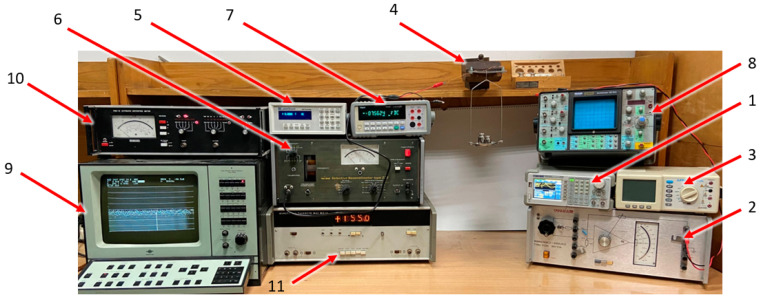
Photo of the test stand with equipment numbered accordingly with the schematic diagram in Figure 4.

Photograph of the measurement system with some auxiliary equipment is presented in Figure 5.

## 3. Results

### 3.1. Power Supply Selection

Using the diagram in Figure 4, the influence of changes in the peak-to-peak amplitude of the voltage applied on the functional generator on the hysteresis error was investigated. In order to determine better method of magnetizing power supply (with current or voltage), the shape of the voltage from the generator (i.e., sinus) and the frequency of 100 Hz remained unchanged.

2.5 Vpp and 5 Vpp amplitudes were tested for the board loads in the range 0–500 g.

The observed uncertainty along with the smaller maximum hysteresis error for the 2.5 V amplitude suggest that the lower peak-to-peak amplitude of the supply voltage applied to the magnetizing coil contributes to the higher accuracy of the converter. Moreover, for 5 V it takes a relatively long time to stabilize the readings, and when the system was left under load, a significant drift of the measured value was observed, probably due to change in magnetizing current in heated coil. For 2.5 V power supply, the maximum hysteresis was 5.01% and for 5 V it was 11.42%. Thus, the 2.5 V power supply would be preferred.

To test the second method of supplying the magnetizing coil, the power generator was replaced with voltage-current converter. In order to power the coil magnetizing the plate, a sinusoidal signal with a frequency of 100 Hz was used with 1.75 A amplitude.

The hysteresis errors were examined, and the duration of measurements and the drift of the measured values were compared. To compare the accuracy of the measurements, it is enough to analyze the average measurement hysteresis

For the current-controlled supply, the largest hysteresis error was slightly over 5%. The measurement process turned out to be much smoother and easier to implement. The drift ceased to be noticeable to such a large extent, and the measurement hysteresis was slightly smaller, therefore current-controlled power supply is recommended instead of voltage controlled one.

### 3.2. THD Measurement

**Figure 6:**
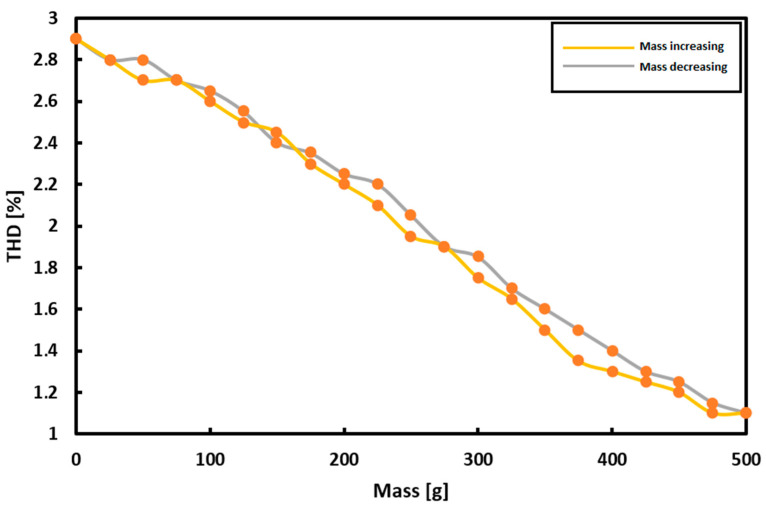
Measurement results of THD of output signal.

The THD of the output signal read from the measuring coil was investigated. The measurement results are presented by the hysteresis loop in Figure 6.

The measurement hysteresis error of over 8% is noticeable. It is worth noting however that the characteristic is almost linear.

### 3.3. Phase Shift Measurement

**Figure 7:**
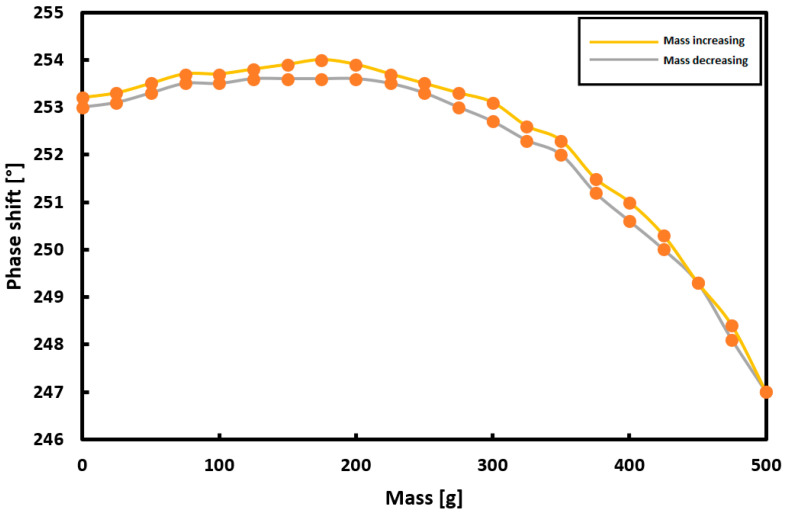
The results of measuring the phase shift between the set and the measured signal for a variable load on the plate.

Measurements of the phase shift between the signal applied to the magnetizing coil and the signal induced in the measuring coil were performed. The measurement results are presented in Figure 7. As for the measurement of THD, the phase shift has a small range of values. The difference between the phase shift for a load of 0 g and a mass of 500 g is only 6.2°. The maximum measurement hysteresis error was 5.71%. The characteristic is unfortunately non-monotonic, and thus cannot be used for sensing applications.

### 3.4. Magnetic Flux Measurement

**Figure 8:**
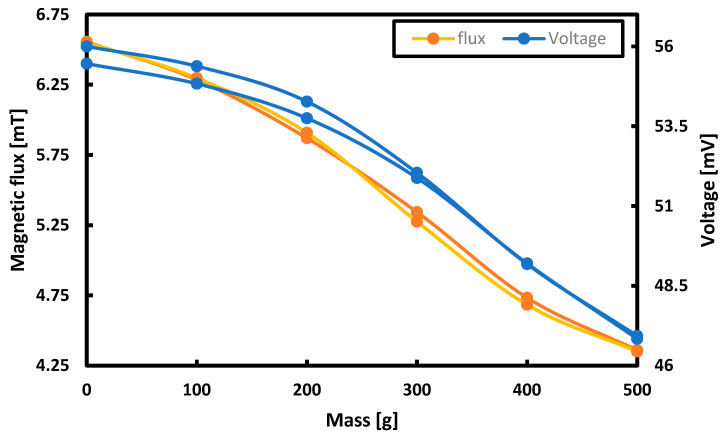
The results of measurements of voltage and magnetic flux for successive masses loading the tested plate.

The quantity directly related to the Villari effect (i.e., changes of the magnetic flux depending on the load) was investigated. The results are presented in Figure 8.

According to the measurements, the maximum hysteresis error for the flux measurement was 3.08% while for induced voltage it was −6%. It was noticed that with the increase of the hysteresis errors for the flux, the hysteresis errors for the voltage measurements decreased.

### 3.5. Harmonics Measurement

A sine function with a frequency of 100 Hz was applied on the function generator to obtain a constant plate magnetizing current of 1.75 A. Using the diagram in Figure 6, measurements were made for the plate loads in the range of 0–500 g with a step of 100 g to the seventh harmonic of the driving current waveform.

**Figure 9:**
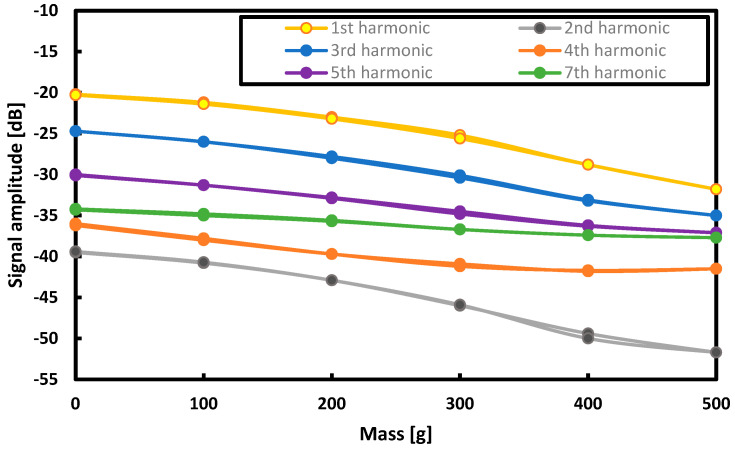
The results of the measurement of the signal amplification measured at the given loads for successive harmonics.

The results are shown in Figure 9. The measurement of the 6th harmonic was dismissed due to the overshadowing by the 7th harmonic.

Despite the schematic nature of the gains of successive harmonics for the second harmonic, the difference in gain between the smallest and largest loads is greater than for any other (including the first). Thus, a series of measurements was made for the second harmonic.

**Figure 10:**
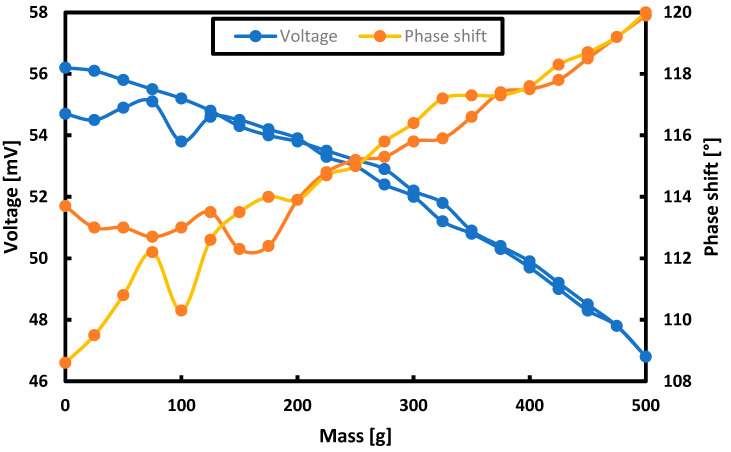
Measurement results of the voltage induced on the measuring coils and the phase shift of the measured signal against the set one in the second harmonic for different masses loading the plate.

Using the same values of the supply current (i.e., 100 Hz), the dependence of the voltage induced on the measuring turns and the phase shift of the induced signal in relation to the signal set on the magnetizing turns was measured for the second harmonic. The results are presented in Figure 10.

The measurement results became less repeatable, and the measurement hysteresis error reached 15.96% for the voltage measurement and 44.74% for the phase shift measurement.

Based on the graphs, it can be concluded that in the second harmonic, the voltage measurement begins to have hysteresis errors comparable to the errors for the first harmonic only after the load exceeds 100 g, and the measurement of the phase shift in the second harmonic cannot be considered more effective than the measurement in the first harmonic. Moreover, it is worth noting that a very large drift of the measured values was observed, which resulted in such large discrepancies in the indications for the same loading mass during increasing and decreasing the load.

**Figure 11:**
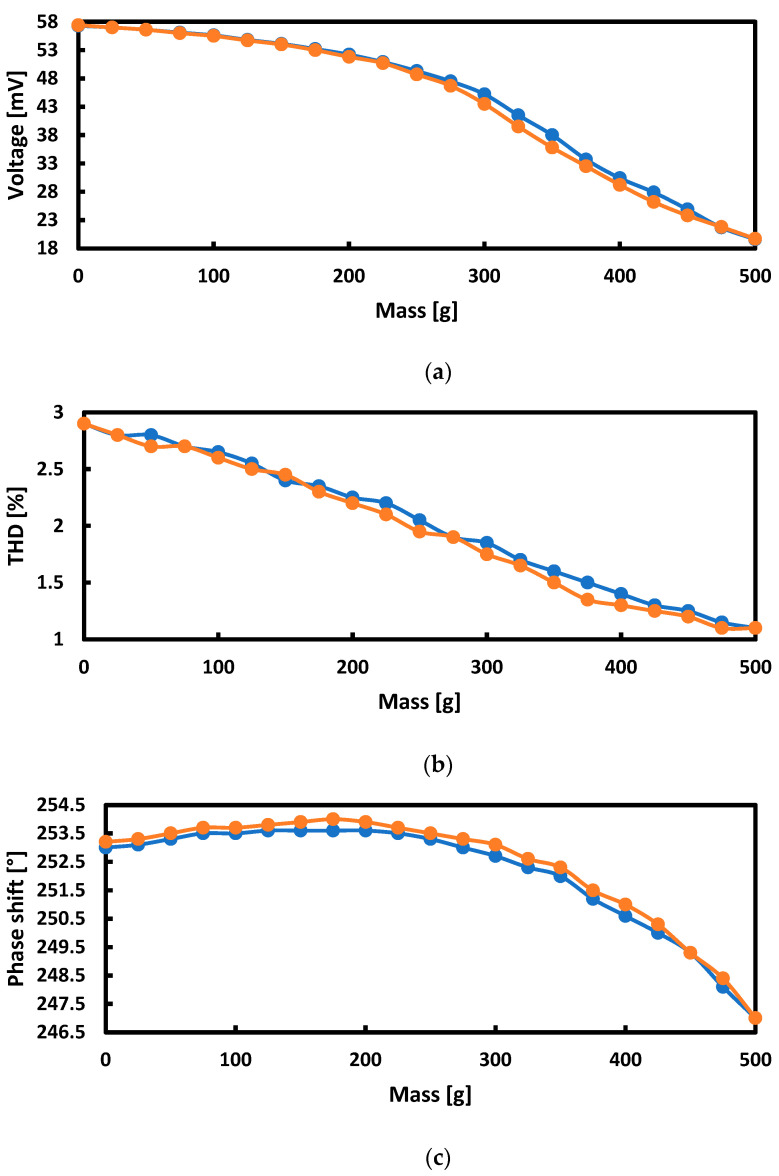

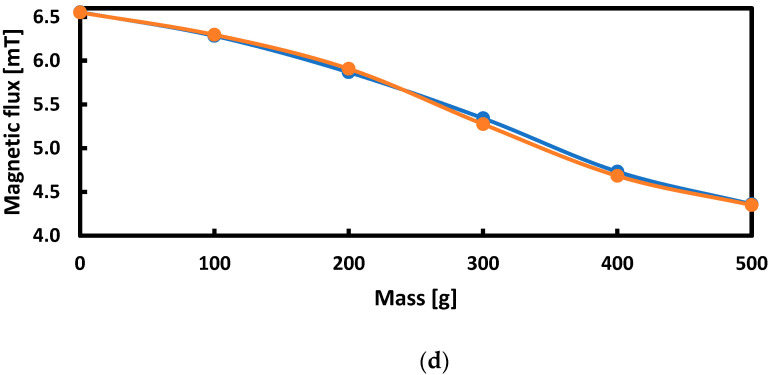
Hysteresis curves for: (**a**) voltage, (**b**) noise, (**c**) phase shift, and (**d**) magnetic flux measurements depending on the weight loading the plate.

As a result of the research, it was decided to consider the measurements in successive harmonics as less effective and to continue with the measurement of the first harmonic only. Figure 11 summarizes the hysteresis curves for the first harmonic for the voltage, THD, phase shift and magnetic flux measurements.

The measurement hysteresis curves turned out to be the narrowest and the least unpredictable for the measurements of the voltage induced in the measuring coils and the magnetic flux.

An interesting perspective, which was not used in further research, may be the fact that the graph of the dependence of THD on the load can be considered approximately linear.

### 3.6. Selection of the Frequency and Shape of the Voltage for the Magnetizing Current

The frequency and shape of the voltage for the magnetizing current for which the hysteresis error was the smallest were determined.

**Figure 12:**
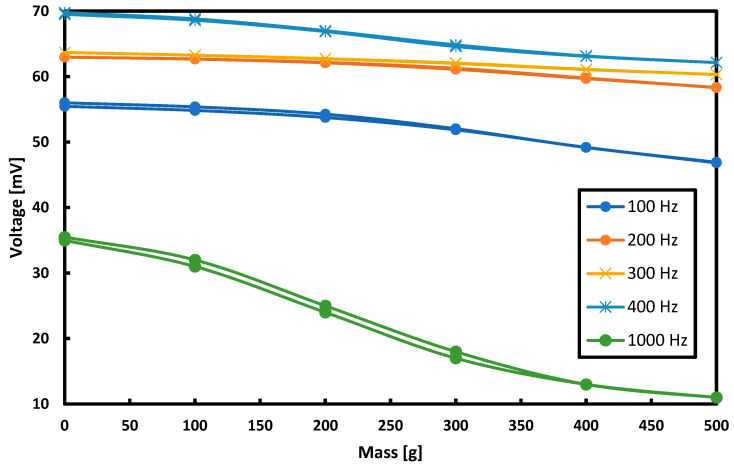
Dependence of the induced voltage on measuring coil on the load for different frequencies of the supply current.

**Figure 13:**
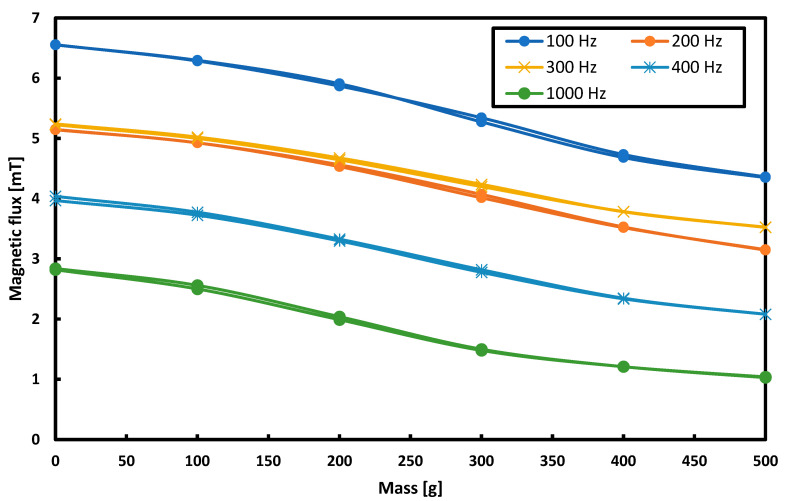
The dependence of the magnetic flux changes on the load for different frequencies of the supply current.

The research started with the current supplying the magnetizing coils with a sinusoidal waveform shape and a constant amplitude of 1.75 A. The effect of changing the frequency of this waveform in the frequency range from 50 to 1000 Hz was measured while keeping the current amplitude constant. The collected results are shown in Figure 12 for voltage measurements and in Figure 13 for measurements of magnetic flux changes.

**Figure 14:**
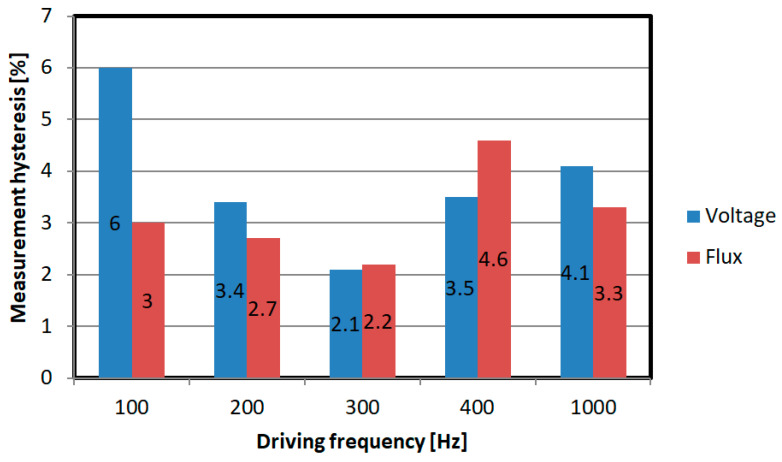
Comparison of maximum hysteresis errors depending on the frequency of the plate magnetizing current.

The maximum measurement hysteresis errors for different frequencies of the sine function are shown in Figure 14.

**Figure 15:**
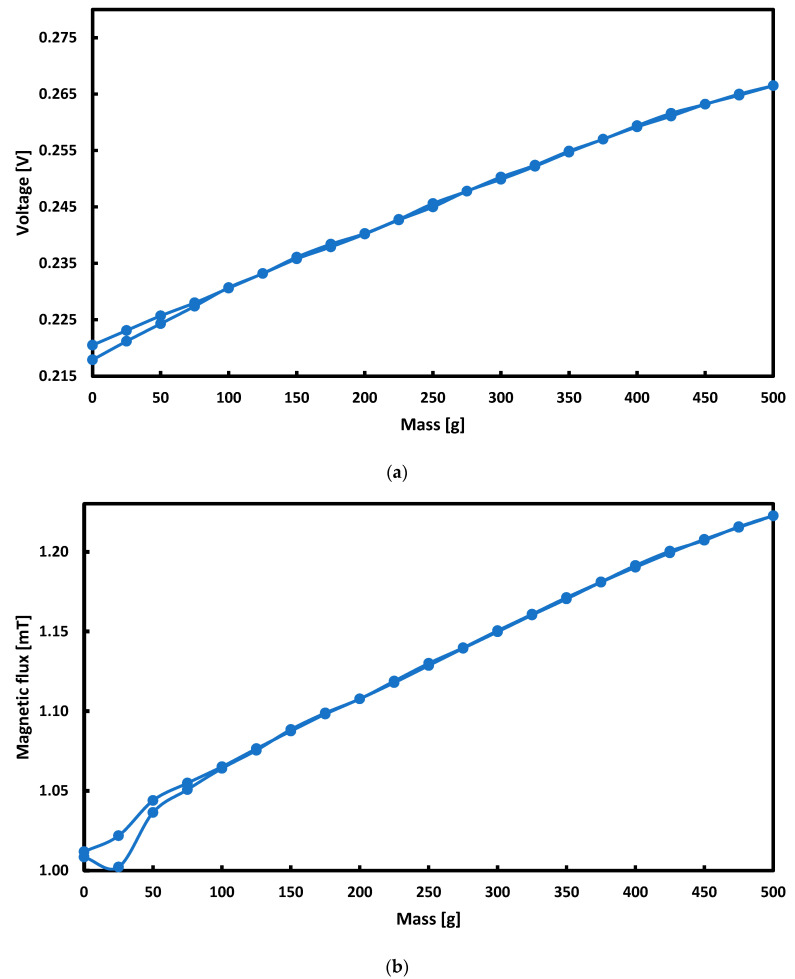
Measurement hysteresis curves for measuring: (**a**) voltage, (**b**) magnetic flux when magnetizing the plate with a triangular signal with a frequency of 300 Hz.

The frequency for which the smallest hysteresis error was obtained for the sinusoidal signal was 300 Hz, both for voltage and flux measurements. For this frequency, the hysteresis error was re-examined for the triangular signal with the current at 1.75 A. The results of these tests are presented in Figure 15.

Based on the above graphs, for the measuring range of 100–500 g loads, the hysteresis error is negligibly small and close to zero (the maximum hysteresis error is 0.65% for the magnetic flux measurement and 1.23% for the voltage measurement). The power supply with a triangular current signal is more effective than a sinusoidal signal, assuming that the lower measuring range is defined as 1 N.

The proposed sensor working in easier to implement voltage mode has following characteristics:

Resolution: 0.01 N (0.2% full range) for voltmeter employed in the tests,

Accuracy: 0.05 N (1% full range), if compensated for non-linearity and hysteresis is considered separately,

Precision: 2 × σ = 0.02 N (0.4% full range),

Non-linearity: 0.25 N (5% full range), can be compensated with higher order polynomial fit to 0.5%,

Measurement hysteresis: 0.1 N (2% full range), which is the most detrimental and hard to compensate source of error, due to magnetoelastic hysteresis.

Total uncertainty is thus assessed, rounding up, as 0.11 N in 1–5 N measurement range.

The signal to noise ratio (SNR) was calculated for the most easily utilized instance, (i.e., voltage signal obtained with 300 Hz magnetizing sinusoidal current (Figure 15a)). It was calculated according to the SNR formula:

$$SNR_{dB}=10log{\left(\frac{A_{signal}}{A_{noise}}\right)}^{2}$$

where:

*SNR_db_* is the signal to noise ratio in decibels, *A_signal_* is the RMS (root mean square) amplitude of measured voltage signal, and *A_noise_* is the RMS amplitude of measured voltage noise.

In the presented case, for *A_noise_* = 1 mV, SNR varies from 46 to 48 decibels, which is far less then the observed measurement hysteresis.

To combat the magnetomechanical hysteresis of the transducer core, it was proposed that amplitude modulated magnetizing current could be better, causing periodic demagnetization of the core.

The following supply waveforms were tested:

- 1 kHz sine modulated with 100 Hz sine—AM,
- 1 kHz sine modulated with 200 Hz sine—AM,
- 1 kHz triangle modulated with 100 Hz sine—AM,
- 1 kHz triangle modulated with 200 Hz sine—AM.

**Figure 16:**
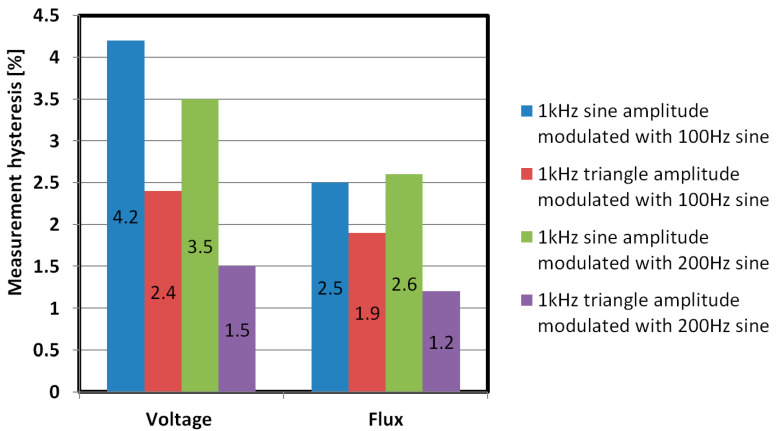
Measurement hysteresis errors for voltage and magnetic flux measurements with an AM modulated magnetizing current supply.

The obtained characteristics were similar to Figure 15a, and measurement hysteresis error values are presented in Figure 16. These were unfortunately higher than for pure triangular signal, and as such are not suitable for sensor operation.

## 4. Conclusions

The designed magnetomechanical force sensor has been tested in the operating range from 0 to ~5 N (0 to 500 g load). Based on these tests, its measuring range can be defined as 1–5 N due to measurement hysteresis in 0–1 N range, but it is potentially scalable with core thickness. The force measurement uncertainty was 0.11 N. The SNR was about 46 dB for voltage signal with the smallest measurement hysteresis. Taking into account the parameters of the sensor, the biggest advantages are its robustness (solid steel as sensing element), high SNR, and the cost of its production mainly due to simple construction and substitution of costly amorphous alloys for popular electrical steel as the sensors core.

The implementation of the core made of transformer steel allows the sensor to be used in difficult operating conditions such as extreme temperatures, humidity, or radiation [3,30]. Various possible methods of effecting the pressure on the sensor core, and variation in its geometrical dimensions allow one to obtain different measuring ranges; there are pressductors on the market with a range of up to 10^6^ N [3,30].

The newly developed sensor works for ranges much smaller and with higher sensitivity than the other magnetoelastic sensors proposed previously. This is mainly due to higher stresses generated in sensors core in bending mode than for, e.g., compressive or tensile stresses for the same force and core dimensions [30]. Additionally, electrical steel proved to have high stress sensitivity of magnetic permeability, which is crucial for sensor construction but may be detrimental in transformer cores design and operation.

## Data Availability

Not applicable.
